# Analysis of two cooperating antibodies unveils immune pressure imposed on HIV Env to elicit a V3-glycan supersite broadly neutralizing antibody lineage

**DOI:** 10.3389/fimmu.2022.962939

**Published:** 2022-09-26

**Authors:** Maxwell T. Finkelstein, Emma Parker Miller, Molly C. Erdman, Daniela Fera

**Affiliations:** Department of Chemistry and Biochemistry, Swarthmore College, Swarthmore, PA, United States

**Keywords:** human immunodeficiency virus (HIV), antibody, Env, crystal structure, negative stain EM, variable loops, glycan

## Abstract

Elicitation of broadly neutralizing antibodies (bnAbs) is a goal of vaccine design as a strategy for targeting highly divergent strains of HIV-1. Current HIV-1 vaccine design efforts seek to elicit bnAbs by first eliciting their precursors through prime-boost regimens. This requires an understanding of the co-evolution between viruses and antibodies. Towards this goal, we have analyzed two cooperating antibodies, DH475 and DH272, which exerted pressure on the HIV population in an infected donor, called CH848, to evolve in such a way that it became sensitive to the V3-glycan supersite DH270 bnAb lineage. We obtained a 2.90Å crystal structure of DH475 in complex with the Man_9_ glycan and a negative stain EM model of DH272 in complex with the HIV-1 spike trimer, Env. Coupled with additional modeling studies and biochemical data, our studies reveal that DH475 contacts a V3- and V4-glycan dependent epitope accessible on an open or shed Env and that DH272 makes critical contacts with the V1V2 and V3 loops on HIV-1 Env. Using these data, we suggest a prime-boost regimen that may facilitate the initiation of DH270-like bnAb precursors.

## Introduction

Current progress towards an effective HIV-1 vaccine is hindered by multiple characteristics of the HIV-1 envelope trimer (Env), a heterodimer of gp120 and gp41 subunits (1). During viral entry, Env undergoes drastic conformational changes. Upon binding to the CD4 receptor, the Env variable loops V1V2 and V3 rearrange leading to an open trimer (2). Env can then bind to a co-receptor that eventually leads to more conformational changes and the shedding of gp120 subunits. Env hinders immune recognition by being heavily glycosylated, conformationally dynamic, and highly sequence variable, particularly at its five variable loops (3–6). Fortunately, broadly neutralizing antibodies (bnAbs) develop in 15% of individuals with HIV and can neutralize many viral variants, making their elicitation a major goal for vaccine design (7). Known bnAbs often have unique features like high levels of somatic hypermutation (SHM) and long complementarity determining region (CDR) loops, which result from long maturation pathways (8). Studying bnAb maturation and their co-evolution with Env is necessary to inform vaccine design strategies, wherein guided immunogen formulations would quickly facilitate bnAb evolution to develop a protective immune response (9).

Cooperating antibody lineages exert evolutionary pressure on HIV, forcing the virus to develop escape mutations in such a way that makes it more sensitive to a bnAb lineage (9). This enables cooperating lineages to facilitate the process of bnAb development (10). Cooperating antibodies are hypothesized to work by targeting the same site on the spike in different ways from a given bnAb lineage. Despite HIV-1/bnAb co-evolution studies against epitopes across Env, only a few cooperating antibody lineages have been identified and structurally characterized (10, 11). In donor CH505, the CH103 and CH235 lineages target the CD4-binding site of gp120, and cooperated with each other to stimulate both lineages to potent neutralization breadth. These findings facilitated the development of a sequential immunization regimen that successfully elicited durable immune responses in non-human primates, but were unable to facilitate bnAb development perhaps due to the restricted orientation of bnAbs that target the CD4-binding site (12–14).

BnAbs against the V3-glycan supersite, on the other hand, can bind to an epitope on Env that includes the N332 glycan in different ways, making their elicitation by vaccination desirable. Donor CH848 developed the DH270 bnAb lineage, which targets this epitope (15, 16), and DH270 lineage development relied on cooperating lineages DH272 and DH475. DH272 and DH475 were able to neutralize autologous virions isolated during the first year of infection, but then lost potency due to mutations in Env. Following this, virions became sensitive to neutralization by DH270 lineage members. CH848 viruses from before this time point had significantly longer V1V2 loops and accumulated other mutations to become sensitive to the DH270 lineage, likely in response to immune pressure by DH475 and/or DH272 (15).

Here we investigate the binding modes of the DH475 and DH272 cooperating antibodies to Env, whose binding modes on Env and influence on viral evolution are currently unknown. In this study we determined a 2.90Å crystal structure of the DH475 Fab in complex with a Man_9_ glycan described previously (17), and a negative stain EM (nsEM) model of a DH272 variant bound to a CH848 Env trimer. We also used molecular modeling, and site-directed mutagenesis coupled with biolayer interferometry (BLI) to characterize how these cooperating antibodies bind Env. Our structural models account for several mutations observed during viral evolution in CH848 that were likely critical in the initiation of the DH270 bnAb lineage and suggest new avenues for further experimentation and investigation to better understand the antibody-virus co-evolution process in the CH848 donor.

## Results

### DH475 binds the N332 glycan of an open or shed Env

**Figure 1:**
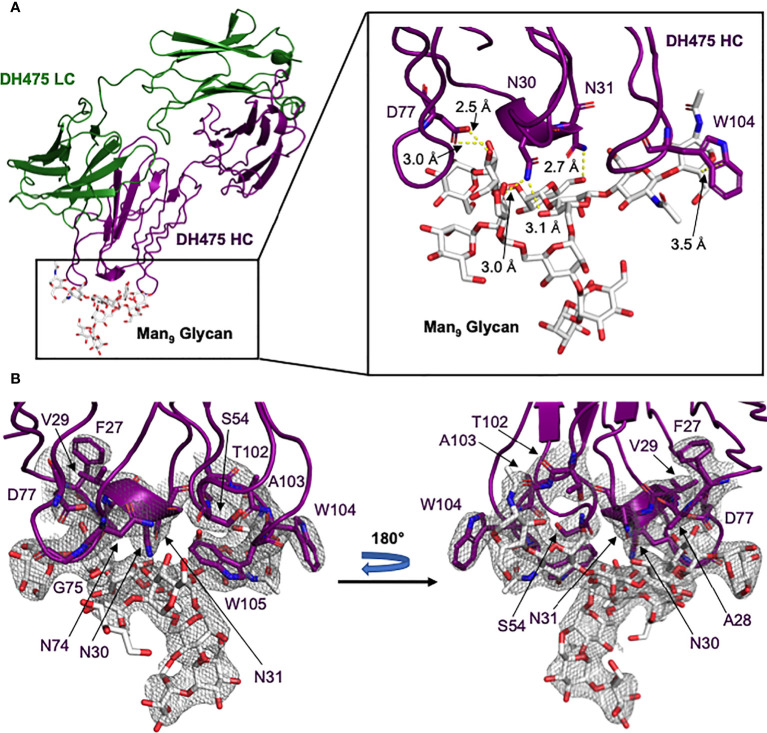
Crystal structure of the DH475-Man_9_ complex. **(A)** The DH475 light chain is shown in green, while the heavy chain (purple) interacts with Man_9_ (white). A zoomed panel is shown on the right, with DH475 heavy chain residues that form key interactions (yellow) with Man_9_ indicated along with their distances. **(B)** The electron density around the heavy chain paratope is shown, with the paratope and Man_9_ shown as sticks and colored by atom (N: blue; O: red).

To determine how DH475 binds Env, we determined a 2.90Å crystal structure of DH475 with a Man_9_ glycan (**Figure 1A**; **Supplementary Table 1**), and it revealed a heavy chain paratope. This paratope was dominated by contacts from a ^27^FAVNN^31^ motif on CDRH1, a ^102^TAWW^105^ motif on CDRH3, and residues N74, G75, and D77 on FW3 (**Figure 1B**). The S54 backbone on CDRH2 also participates in this interaction. On CDRH1, N30 and N31 both form hydrogen bonds with mannose moieties of the pentasaccharide core of Man_9_, and N30 forms an additional hydrogen bond with mannose B on the Man_9_ D3 arm (**Figure 1A**). W104 on CDRH3 forms a hydrophobic stacking interaction with the core N-acetyl glucosamine, while D77 on FW3 forms two hydrogen bonds with mannose C on the Man_9_ D1 arm. While the D1 and D3 arms of Man_9_ interact with the DH475 heavy chain, the D2 arm is distal to the binding interface.

To evaluate the physiological relevance of the DH475-Man_9_ crystal structure, we introduced mutations into DH475 and tested their effects on binding to Env using BLI. Specifically, we generated N30A, N31A, D77A, or W104A DH475 heavy chain mutants and assessed their binding to the CH848 transmitted/founder (TF) gp120 core (**Table 1**). Wild-type DH475 bound to the CH848 TF gp120 core with a K_D_ of 4.0µM, and mutation of the paratope residues eliminated binding affinity (K_D_>50µM), confirming the physiological relevance of the DH475-Man_9_ crystal structure.

**Table 1:** Dissociation constants (µM) between cooperating antibodies and CH848 TF Env constructs determined using biolayer interferometry.

	gp120 core	N413A gp120 core	gp120 core mV5	N462A gp120 core	N464aA gp120 core	DS SOSIP chimera	N332A DS SOSIP chimera	Y173A DS SOSIP chimera	D321A DS SOSIP chimera
WT DH475	4.0	14	5.2	2.9	3.3	–	–	–	–
N30_H_A DH475	>50	–	–	–	–	–	–	–	–
N31_H_A DH475	>50	–	–	–	–	–	–	–	–
D77_H_A DH475	>50	–	–	–	–	–	–	–	–
W104_H_A DH475	>50	–	–	–	–	–	–	–	–
DH272.2	–	–	–	–	–	3.5	5.0	25	~50

**Figure 2:**
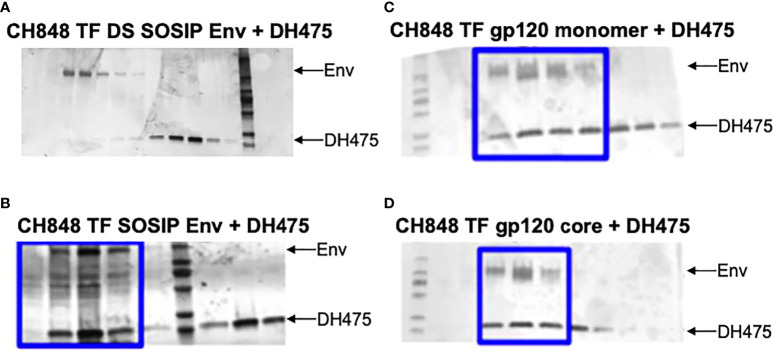
SDS-PAGE gels of co-elution experiments between DH475 and CH848 Envs. DH475 was incubated with the **(A)** CH848 TF DS SOSIP, a closed Env trimer, **(B)** CH848 TF SOSIP, an open Env trimer, **(C)** CH848 TF gp120 monomer and **(D)** the CH848 TF gp120 core which has a V1V2 loop deletion and V3 loop truncation. Fractions containing both DH475 and the Env species are indicated with a blue box.

To determine the binding mode of DH475 on an Env trimer, we first performed co-elution experiments using size exclusion chromatography with different CH848 TF Envs. DH475 was unable to form a stable complex with the CH848 TF DS SOSIP Env trimer which was locked in the closed conformation with two disulfide bonds (**Figure 2A**), but was able to co-elute with the CH848 TF SOSIP Env trimer that could potentially shift between the closed and open conformations (**Figure 2B**). Consistent with this, DH475 was also found to co-elute with a monomeric CH848 TF gp120 and a CH848 TF gp120 core, which has a V1V2 deletion and V3 loop truncation (**Figures 2C, D**; **Supplementary Figure 1**). Taken together, these co-elutions indicate that DH475 can bind to either an open CH848 TF Env trimer, or to a gp120 subunit that has shed, and that the V1V2 loops are dispensable for DH475 binding.

**Figure 3:**
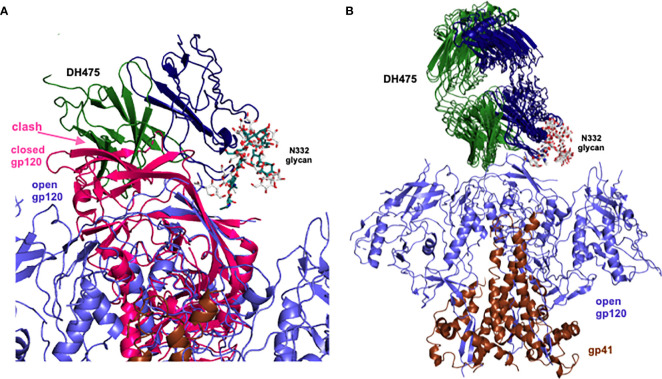
Superpositions of Man_9_ of the DH475 complex onto the N332 glycan of Env. **(A)** Superposed DH475 (heavy chain: blue, light chain: green), closed gp120 (PDB ID: 5W6D, pink) and open gp120 (PDB ID: 7LOK, purple) are shown. **(B)** Successful superpositions (PDB IDs: 4R2G, 5W6D, 5FYK, 5T3Z, 5T3X) of an open Env trimer (gp120: purple, gp41: brown) bound to manually superposed DH475 (heavy chain: blue, light chain: green). Orientation of DH475 relative to Env is the same as that in **(A)**.

To visualize a structure of the DH475-Env trimer complex, we manually superposed Man_9_ in the DH475-Man_9_ crystal structure onto N332 Man_9_ of open and closed Env trimers, since it has been previously shown that DH475 depends on the N332 glycan for binding and neutralization (15). Additionally, we focused our alignments on the core sugar moieties of Man_9_, as well as regions interacting with the DH475 heavy chain in the DH475-Man_9_ crystal structure (**Figure 1**). Specifically, manual superpositions were performed using the second N-acetyl glucosamine and mannose 3 of Man_9_, as well as mannose 4 and mannose C on the Man_9_ D1 arm which interact with N31 and D77 on the DH475 heavy chain, respectively. Superposition onto five of the published structures identified a binding mode in which DH475 may bind an open Env gp120 but clashes with the V1V2 loops of a closed gp120 (**Figure 3A**), consistent with co-elution data showing DH475 formed a stable complex with an open Env trimer or gp120 monomer, but not a closed Env trimer (**Figure 2**). These superpositions yielded similar approaches of DH475 on Env (**Figure 3B**), suggesting a single DH475 binding mode.

### DH475 requires a V4 loop glycan epitope, and not a V5 glycan epitope

Because previous work showed that viral resistance to DH475 increased 5-fold when a V1 deletion was combined with a deletion in the V5 loop, or with a mutation in V4 which ablated a glycosylation site (15), we wanted to next determine if the Env V4 or V5 loops were critical for the DH475 interaction. Our analysis of CH848 Env sequences indicated a potential role of the N413 glycan (HXB2 numbering) on the V4 loop for DH475 binding. This glycosylation site is seldom found in viral isolates besides CH848. T415, which is part of the same N-X-S/T glycosylation sequon as N413, was present across CH848 Env isolates, whereas N413 was present on the CH848 TF Env and became mutated during the first year of infection. Indeed, this region of V4 could be contacted by DH475 in our model, and introducing the N413Y mutation in a CH848 Env isolated at week 39 post-infection was sufficient for removing neutralization ability by DH475 (15). While mutations in regions of Env outside of the variable loops present in CH848 sequences analyzed from the 5-year infection period may have also affected DH475 binding, a comparison of DH475 binding affinity to CH848 Envs indicates significant reductions when the N413 glycosylation site was removed (P<0.001) (**Supplementary Figure 2**). This suggests a potential role of the N413 glycan in mediating DH475 binding to Env, and DH475 may have selected for mutation of this site during HIV-1 infection of donor CH848. To test this, we removed this glycosylation site in the CH848 TF gp120 core and tested binding by BLI. We observed reduced binding to DH475 by the mutant (K_D_=14µM compared to 4µM for the wild-type core) (**Table 1**). This suggests a role for the CH848 Env V4 loop for DH475 binding, consistent with previous observations (15).

Since DH475 was demonstrated to contact the N332 glycan on the V3 loop and given the large distance between the V3 and V5 loops on Env (**Supplementary Figure 3**), we postulated that if any interactions with the V5 loop existed, they would occur through a V5 loop glycan. To test this, we generated a CH848 TF gp120 core mV5 construct, which has identical sequence to CH848 TF gp120 core but with a truncated V5 loop (**Supplementary Figure 1**). Truncation of the V5 loop disrupted two potential N-glycosylation sequons at N462 and N464a in the wild-type sequence. BLI data indicated that there was not a significant reduction in the affinity of DH475 for the CH848 TF gp120 core mV5 (K_D_=5.2µM) compared to the CH848 TF gp120 core (K_D_=4.0µM) (**Table 1**). DH475 was also able to bind both single mutant CH848 TF gp120 core constructs with similar affinities (K_D_=2.9µM and K_D_=3.3µM) (**Table 1**). This suggests that DH475 does not require either glycan on the V5 loop for binding. While this does not preclude DH475 from making contact with V5 glycans, interactions with the V5 loop are unlikely to be critical for DH475 binding.

### DH272 contacts both the V1V2 and V3 loops of Env

**Figure 4:**
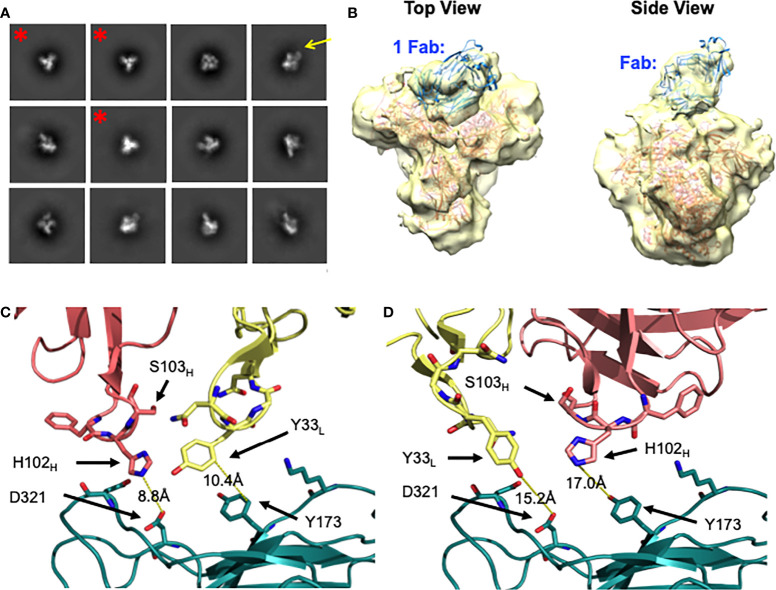
Negative stain EM of DH272.2 - CH848 TF DS SOSIP Env complex. **(A)** 2D class averages of DH272.2 bound to CH848 TF DS SOSIP Env generated from nsEM. Fab density from a select 2D class average is indicated with a yellow arrow. Classes of unliganded Env trimer are indicated with a red asterisk and were excluded from 3D model generation. **(B)** Top and side views of the 3D model of DH272.2 bound to CH848 TF DS SOSIP Env generated from nsEM. The EM map is in transparent yellow and high-resolution structures of CH848.d0949.10.17 DT DS SOSIP trimer (salmon, PDB ID 6UM5) and DH272 Fab (teal, PDB ID 5TRP) fit into the map. **(C)** Close-up view of one orientation of DH272 Fab (heavy chain shown in salmon, light chain shown in yellow) relative to a homology model of CH848 TF gp120 (shown in teal and aligned to CH848.d0949.10.17 gp120, not shown). **(D)** Close-up view of another orientation of DH272 Fab, rotated 180 degrees relative to that in **(C)** relative to a homology model of CH848 TF gp120. Color scheme is the same as in **(C)**. Residues on or near the interface, potentially involved in binding interactions, are shown as sticks.

DH272 was found to bind the CH848 TF SOSIP Env with weak affinity (data not shown) and so we worked with a related antibody, called DH272.1, which had the same light chain but different heavy chain from DH272. The DH272.1 Fab had higher affinity to the CH848 TF SOSIP Env and to alter binding, we introduced the D102_H_H and G103_H_S mutations to produce DH272.2 (**Supplementary Figure 4**). These mutations were reversion mutations to residues found in DH272 and improved binding to Env (K_D_ = 3.5μM versus 11.5μM), as determined using BLI. Using this mutant, we determined a 14.4 Å nsEM 3D model with the CH848 TF DS SOSIP Env trimer (**Figure 4**). The model shows that a DH272.2 Fab binds the trimer and contacts both the V3 region, like DH270, as well as the V1V2 loops. Only one Fab was visibly bound to our trimer from nsEM, though multiple Fabs were observed to bind Env in some of our nsEM images (**Supplementary Figure 5**). To determine the number of DH272.2 Fabs that could bind the CH848 TF DS SOSIP Env in solution, we performed ITC and obtained a binding stoichiometry of 3:1 (**Supplementary Figure 6A**). To determine if three Fabs could indeed bind without steric interference based on the binding site observed in our EM map, we aligned three copies of the DH272.2 – CH848 TF DS SOSIP Env complex map such that each gp120 monomer would be bound to a single Fab. This superposition revealed that there would be no clashing between neighboring Fabs or monomers (**Supplementary Figure 6B**), demonstrating that three Fabs could indeed bind simultaneously to Env.

To analyze the binding between DH272 and the CH848 TF Env, we developed a homology model of the CH848 TF Env using the known cryo-EM structure of CH848.d0949.10.17 DT DS SOSIP Env (16). We then manually docked this homology model, along with the crystal structure of DH272 (15), in the nsEM map (**Figures 4B-D**). Due to the limited resolution of our nsEM map, we identified two possible orientations of DH272 binding to the V1V2 and V3 loop regions of Env and examined both orientations to inform further biochemical analyses. To identify important points of contact at the interface between DH272 and CH848 TF Env, we introduced mutations at residues of interest to determine whether such changes would alter binding. First, we noticed from our models that, unlike the DH270 bnAb lineage with which DH272 cooperates, the Fab does not appear close enough to interact with the N332 glycan. To test this prediction from our models, we introduced the N332A mutation into the CH848 TF DS SOSIP Env chimera (with the gp41 region of BG505 and whose K_D_ of binding to DH272.2 was the same as that of the CH848 TF DS SOSIP Env (data not shown)) to remove glycosylation at this site and observe any changes in binding. Using BLI, a K_D_ value of 5.0μM was obtained for the mutant binding to DH272.2, compared to 3.5μM with the wild-type (**Table 1**). These results indicate that no substantial change in binding was caused by the removal of the N332 glycan.

Based on our molecular modeling, we also identified two amino acid residues on the CH848 TF Env, Y173 and D321, that might contribute to binding DH272 (**Figure 4**). We mutated each of these residues to alanine to eliminate any possible hydrophobic stacking, hydrogen bonding, or salt bridge interactions and tested binding to DH272.2 by BLI. For both mutants, we saw a substantial decrease in binding: the CH848 TF DS SOSIP Env chimera Y173A mutant had a K_D_ of 24.9μM (**Table 1**), while the CH848 TF DS SOSIP Env chimera D321A mutant had a K_D_ of ~50μM (**Table 1**). Interestingly, Y173A is located on the V1V2 loops of Env, while D321 is located on the V3 loop, confirming that DH272 binds both of these regions. Together, they make up an epitope largely distinct from that of DH270, with a small amount of overlap at the D321 residue on the V3 loop.

## Discussion

Understanding how cooperating antibodies direct HIV-1 co-evolution with bnAb lineages is crucial to the development of effective HIV vaccines. In donor CH848, multiple regions of Env were mutated throughout five years of infection (15). In the variable loops, this included deletions in V1V2 and V5 and a mutation in the V4 loop (N413) within the first year of infection (i.e., when the virus was sensitive to DH475 and DH272 neutralization), while mutations in V3 occurred later in infection. In this study, we identified key characteristics of the binding modes of these two cooperating antibodies to the CH848 TF Env. DH475 binds a glycan-dependent epitope on an open or shed Env that has already bound CD4 and/or a co-receptor and its binding site spans the V3 loop and the base of the V4 loop. DH272 interacts with the V1V2 and V3 regions of Env.

It was previously shown that DH475 requires the N332 glycan for binding (15) and here we further show a dependence on the N413 V4 glycan from the CH848 TF Env for binding. Taken together, our model indicates that DH475 binds the N332 glycan with a distinct binding approach to previously characterized V3-glycan supersite bnAbs (17–21). Glycan-directed epitopes provide an effective way for antibodies to maintain potent neutralization breadth, since viral evolution to ablate glycosylation sites may increase the immunogenicity of the viral spike. Ablation of the N413 glycan on the V4 loop was sufficient to reduce DH475 binding and may have allowed DH270 lineage members to bind. Analysis of the cryo-EM structure of DH270.6 in complex with Env shows that a glycan at position 413 could potentially sterically interfere with antibody binding, consistent with this hypothesis (16).

In the case of DH272, while the resolution of our nsEM map was insufficient to unambiguously identify the binding orientation of DH272 to Env, our structural and biochemical data revealed that Env residues Y173 and D321 are important for binding, implicating the V1V2 and V3 loops in binding. The dependence on binding the V1V2 region was likely critical in the development of the DH270 bnAb lineage since a large V1V2 deletion occurred before the bnAb lineage could bind Env. The DH270 bnAb precursor, UCA3, even appeared to engage the shorter V1 loop of a later variant of Env, CH848.d0949.10.17 Env, unlike its mature bnAb descendent, DH270.6 (16).

These findings highlight important features of Env that may have supported initial activation of the DH270 unmutated common ancestor (UCA). Notably, while others identified shorter V1V2 loops to associate with DH270 lineage neutralization potency (15), we extend on their findings by identifying this epitope as part of the DH272 binding interface, providing support for the hypothesis that DH272-directed viral evolution facilitated DH270 lineage induction. More significantly, by examining the DH475 binding interface, we are the first to identify the importance of ablation of the N413 glycan during CH848 Env evolution, which may have aided activation of the DH270 UCA.

Both DH475 and DH272 have limited breadth, illustrating the importance of considering antibodies, even if they are not broadly neutralizing, to understand bnAb developmental pathways. The distinct binding modes of DH475 and DH272 also highlight the diversity of antibodies that can be crucial to the development of a single bnAb lineage. Additionally, they have distinct epitopes from the DH270 bnAb lineage and our findings show that cooperating antibodies can exert selection pressure on HIV-1 to support bnAb development with only minor epitope overlaps. Notably, DH272.2 binds Env more similarly to V1V2-directed bnAbs (22–25) than to V3-glycan supersite bnAbs (16, 26–28). Like V3-glycan supersite bnAbs, however, up to three copies of DH272.2 could bind to a trimer, as was observed using ITC. The discrepancy in binding stoichiometry when compared to our nsEM data is likely due to differences in saturation when comparing complexes at equilibrium as in the case of nsEM versus the maximum binding observed using ITC, similar to what was observed by others for VRC38 (22).

While more complete and/or higher resolution models of DH272 and DH475 in complex with Env will likely shed more light on additional critical interactions and their roles in viral-antibody co-evolution, our results suggest some important aspects of prime-boost regimens that might induce the development of DH270-like bnAb precursors. Specifically, if one were to use live attenuated virus vaccines, the viral components would bear a long V1V2 loop on a closed trimeric Env and the N413 glycan on either an open Env or the gp120 subunit alone to expand DH272- and DH475-like cooperating lineages, respectively. These antibodies would then produce Env variants that can trigger DH270-like bnAb precursors. For vaccine types that do not rely on viral replication, Env immunogens would need to contain a shorter V1V2 loop and lack the N413 glycan to expand the desired DH270-like bnAb precursors. Then, Envs that could elicit improbable mutations in DH270-lineage members, as described elsewhere, would be used (16, 17, 29). This regimen would be analogous to the prime-boost regimens previously used for the CH103 and CH235 cooperating bnAb lineages, for which structural investigations guided the development of immunogens that elicited long-lasting antibody responses in macaques (12–14).

## Materials and methods

### Site-directed mutagenesis

Site-directed mutagenesis was performed using a modified manufacturer’s protocol (Stratagene) to introduce mutations into the Env and Fab constructs. Mutagenesis reactions comprised 120ng template DNA, 100ng mutagenic primers, 4.5nmol dNTPs, Pfu reaction buffer (Agilent Technologies), and 1.5U Pfu Turbo DNA polymerase (Agilent Technologies) in separate initial reaction mixtures for the forward and reverse primer, which were subjected to three cycles of initial amplification (95°C 30s, 50 °C 1min, 65°C extension time). Extension times were customized to plasmid length; extension time (min) = 2 × plasmid length (kb) + 1-2 min. Amplified initial reaction mixtures were then mixed, supplemented with 2.5U Pfu Turbo, and the resulting mixture was amplified for eighteen cycles with identical parameters to the initial amplification. Parent template DNA was digested with 10U DpnI (Agilent Technologies) for 1.5hr at 37°C prior to bacterial transformation. Sequencing was confirmed by submitting samples to Azenta Life Sciences.

### Maintenance of mammalian cells

HEK293S GnTI^-^ and HEK293F cells were grown in suspension at 37°C. HEK293F cells were grown in FreeStyle 293 GlutaMAX media (Gibco), supplemented with 100,000U Penicillin-Streptomycin (Gibco) per 1L media. HEK293S GnTI^-^ cells were maintained using similar Freestyle 293 GlutaMAX media with Penicillin-Streptomycin, but with additional supplementation of 2% ultra-low IgG fetal bovine serum.

### Expression and purification of Fabs

DH475 Fabs were produced without tags; DH272 Fabs were produced with His-tags on the heavy chain C-terminus. Fabs were expressed in HEK293F suspension adapted cells using transient transfection with linear polyethylenimine (PEI). For 200mL of cells, a total of 100µg of filtered plasmid DNA was mixed with 500µL PEI in 10mL 1× phosphate buffered saline (PBS), incubated for twenty minutes, and then added to the cell culture. Heavy chain and light chain DNA were added in a 1:1 ratio. Proteins were allowed to express for five days before harvesting. After five days of expression, the cells were centrifuged (3000 RPM for 30 minutes using a Sorvall Legend RT+ Centrifuge), filtered (0.2µm polyethersulfone), and treated with 0.02% sodium azide prior to affinity chromatography.

The supernatant containing DH475 Fabs was diluted twofold in PBS prior to purification. CaptureSelect LC-lambda Affinity Matrix resin (ThermoFisher Scientific) was pre-equilibrated with PBS, and then the diluted supernatant was passed through the column containing the resin for affinity chromatography. After the supernatant finished flowing through, the column was washed with PBS, and eluted with 100mM glycine buffer pH 3.0. The eluate was neutralized with 1M Tris buffer pH 8.0 (by adding to make up 1/10^th^ the volume of the eluate) before concentrating and centrifuging for size exclusion chromatography (SEC). SEC was done using a Superdex 200 Increase 10/300 GL column (Cytiva) in 2.5mM Tris buffer pH 7.5, 350mM NaCl. Fractions of interest were >90% pure, confirmed using SDS-PAGE, and further concentrated before storage at 4°C.

The supernatant containing DH272 Fabs was passed through a Ni-NTA resin affinity column, pre-equilibrated with 10mM Tris pH 7.5, 200mM NaCl. After loading, the column was washed again with 10mM Tris pH 7.5, 200mM NaCl and then with 10mM Tris pH 7.5, 200mM NaCl, 10mM Imidazole. Fab was eluted with 10mM Tris pH 7.5, 200mM NaCl, 350mM Imidazole and then concentrated for size exclusion chromatography (SEC). SEC was run using a Superdex 200 increase column in a buffer of 2.5mM Tris pH 7.5, 350mM NaCl. Fractions containing protein of interest (as determined using SDS-PAGE) were collected and concentrated to be stored at 4°C until further experimentation.

### Expression and purification of Envs

CH848 TF Envs were expressed in HEK293S GnTI^-^ cells *via* transient transfection with linear polyethylenimine (PEI). In the case of gp120 monomeric constructs, 100µg of filtered plasmid DNA was mixed with 500µL PEI in 10mL PBS for 200mL of cells, incubated for twenty minutes, and then added to the cell culture. Proteins were allowed to express for five days before harvesting. In the case of Env trimers, 80μg Env trimer DNA and 20μg furin DNA in 10mL PBS + 0.5mL 1mg/mL PEI was transfected per 200mL cells in suspension. Env was allowed to express for 6–7 days before harvesting.

After expression, cells were centrifuged (3000 RPM for 30 minutes using a Sorvall Legend RT+ Centrifuge) and filtered (0.2µm polyethersulfone) prior to affinity chromatography. Cell supernatant was treated with 0.02% sodium azide and an EDTA-free protease inhibitor tablet. The supernatant was then passed through a *Galanthus nivalis* lectin resin affinity column pre-equilibrated in 1x PBS. In the case of monomeric Envs, after the supernatant finished flowing through, the column was washed with PBS, and eluted with 0.5M methyl-α-D-mannopyranoside dissolved in PBS. Envs were then purified *via* SEC using a Superdex 200 Increase 10/300 GL column (Cytiva) in 2.5mM Tris buffer pH 7.5, 350mM NaCl. In the case of trimeric Envs, after the supernatant flowed through, the column was washed first with 1x PBS and then with 0.5M NaCl dissolved in 1x PBS. Env was eluted with 0.5M methyl alpha-D mannopyranoside dissolved in 1x PBS and then concentrated for SEC. SEC was run using a HiLoad Superdex 200 prep column in a buffer of 5mM Hepes pH 7.5, 150mM NaCl. Fractions of interest contained protein that was >90% pure (which excludes glycan heterogeneity), confirmed using SDS-PAGE, and further concentrated before being flash frozen and stored at -80°C until further experimentation.

### Co-elution binding experiments

50μg of Env was incubated with 5–8x molar excess of Fab in the case of Env trimer or at 1.3-1.4x molar excess Fab in the case of Env monomer in 500μL buffer (typically 2.5mM Tris pH 7.5, 350mM NaCl but in some instances 5mM Hepes pH 7.5, 150mM NaCl was used) and allowed to incubate on ice for 90 minutes. SEC was then run using a Superose 6 increase column (Cytiva) for complexes with trimer or a Superdex 200 increase column (Cytiva) for complexes with Env monomer in the same buffer used for incubation. Fractions with protein were run on SDS-PAGE and visualized with silver stain.

### Biolayer interferometry

Binding was assessed using a BLItz instrument (fortéBIO, Pall Corporation). Dilutions of Fabs Env constructs were made in 2.5mM Tris buffer pH 7.5, 350mM NaCl, and this buffer was used for both baseline measurements and the dissociation step. Fab constructs were diluted to 0.2mg/mL and immobilized onto Anti-Human Fab-CH1 2nd Generation (FAB2G) Biosensors. The Env constructs were titrated to assess binding affinities, with concentrations chosen to maximize fit during K_D_ calculation. Each run comprised a 30 second baseline, followed by 150s of Fab loading, and then 30s baseline before 180s of Env association, and finally 180s of dissociation. Step corrections at the start of association and dissociation were performed using Microsoft Excel to normalize the data for association to the starting value of zero, and data for dissociation was normalized such that the beginning value of dissociation was equal to the ending value of association. Binding parameters were obtained through global fit of each experiment using nonlinear regression curve fitting in Graphpad Prism.

### Isothermal titration calorimetry binding experiments

A Malvern MicroCal PEAQ-ITC System was used to inject 3.4 μL of DH272.2 at a concentration of 157 μM into a cell containing the CH848 TF DS SOSIP Env at a concentration of 7.59 μM. The first injection was 0.4 μL and its data point was later excluded from the analysis. The initial delay was 60 seconds and the delay after the first and subsequent injections were 150 seconds and 180 seconds, respectively. Data analysis was performed using Malvern MicroCal PEAQ-ITC Analysis software, fitting data to a model for single site binding kinetics.

### Crystallization, structure determination, and refinement

Man_9_-biotin and Man_9_-V3-biotin were synthesized as described earlier (30). The DH475 Fab was mixed with 6 molar excess of Man_9_ glycan at a final complex concentration of 25 mg/mL for crystallization trials. Crystals were grown in 96-well format using hanging drop vapor diffusion at 20°C. The complex crystallized over a 100uL reservoir of 1.5 M AmSO_4_ and 100 mM PIPES, pH 6.0 in 1-2 days. Crystals were cryoprotected by brief immersion in reservoir solution supplemented with 25% glycerol before being flash frozen in liquid nitrogen.

X-ray diffraction data was collected using a beamline at the Advanced Photon Source at Argonne National Laboratory. DH475-Man_9_ complex data were collected at beam line 24-ID-C using a single wavelength of 0.98 Å. Data set from an individual crystal was processed with XDS (31). Molecular replacement calculations for the Fab were carried out with PHASER (32) using published DH270.5 [Protein Data Bank (PDB) ID 5TPP] as the search model. The Fab was broken up into the variable and constant domains in order to perform two separate searches. DH475 contained 1 molecule in the asymmetric subunit.

Refinement was carried out using PHENIX (33), with manual modifications carried out using Coot (34). Maps were generated using combinations of positional, individual B factors, and TLS (translation/libration/screw) refinement algorithms. Combinations of secondary structure restraints, optimizations of X-ray/stereochemistry weight, and optimizations of X-ray/ADP weights were used during refinement. Structures were assessed using the MolProbity server (35).

### Negative stain electron microscopy

Purified CH848 TF DS SOSIP Env trimer was incubated with a 6 molar excess of DH272.2 Fab at 4 °C for 1.5 h. A 2.5 µl aliquot containing ∼0.01 mg/ml of the Fab-SOSIP complex was applied for 30 s onto a carbon-coated 400 Cu mesh grid that had been glow discharged at 20 mA for 2 min, followed by negative staining with 0.7% (w/v) uranyl formate for 20 s. Samples were imaged using a FEI Tecnai TF20 microscope operated at 200 kV and a magnification of ×62,000, yielding a pixel size of 3.000 Å at the specimen plane. Images were acquired with a Tietz F416 camera using a nominal defocus of -3000 nm.

Particles were picked automatically using cryoSPARC and put into a particle stack. Initial, reference free, two-dimensional (2D) class averages were calculated, and particles corresponding to complexes (with at least one Fab bound) were selected into a substack for determination of an initial model. The initial ab initio model was calculated in cryoSPARC without symmetry and cryoSPARC was used for subsequent refinement. The resolution of the final model was estimated using a Fourier Shell Correlation (FSC) cut-off of 0.5.

### Protein structure analysis and visualization

Manual superpositions of DH475-Man_9_ onto the N332 glycan of published high-resolution structures of Env constructs were done using PyMOL. Homology models were generated using SWISS-MODEL (36–38) and visualized in PyMOL. nsEM density maps were visualized in UCSF Chimera. The structures were fit into the nsEM density maps by first placing the Env and two orientations of the Fab into the map manually and then using the UCSF Chimera ‘Fit in map’ function. Once fitted, relative orientations of high-resolution Fabs and Envs, including residues at the interface, were visualized in PyMOL. Figures were generated from PyMOL and UCSF Chimera.

## Data availability statement

The datasets presented in this study can be found in online repositories. The names of the repository/repositories and accession number(s) can be found below: Protein Data Bank with accession code 8D3A and EM Data Bank with accession code EMD-28120.

## Author contributions

DF coordinated and designed the study. MF, EPM, ME and DF, analyzed and evaluated the data, and wrote and edited the manuscript and figures. MF. EPM, ME and DF performed experiments. MF, EPM, and ME and DF expressed and purified proteins. DF crystallized complex, collected diffraction data and solved structure. MF refined structure and generated mutations. DF prepared nsEM samples. EPM analyzed EM data and generated mutations. MF and EPM performed BLI experiments. All authors contributed to the article and approved the submitted version.

## Funding

This research was supported by the National Institute of Allergy & Infectious Disease NIH award 1R15AI150484 to DF. MF was supported by the Swarthmore College Honors Fellowship, ME and EPM were supported by the Frances Velay Fellowship through the Panaphil Foundation, and EM was also supported by the Swarthmore College Mayer Davidson funds.

## Acknowledgments

We thank the Haynes Laboratory at the Duke University School of Medicine for providing sequences of DH272 related antibodies. We thank staff at Northeastern Collaborative Access Team (NE-CAT) X-Ray beam line 24 ID-C (Advanced Photon Source). This work is based upon research conducted at the Northeastern Collaborative Access Team beamlines, which are funded by the National Institute of General Medical Sciences from the National Institutes of Health (P30 GM124165). This research used resources of the Advanced Photon Source, a U.S. Department of Energy (DOE) Office of Science User Facility operated for the DOE Office of Science by Argonne National Laboratory under Contract No. DE-AC02-06CH11357. We also thank the staff at the National Center for CryoEM Access and Training for their assistance with collecting nsEM data of DH272.2 bound to CH848 TF DS SOSIP Env. Some of this work was performed at the National Center for CryoEM Access and Training (NCCAT) and the Simons Electron Microscopy Center located at the New York Structural Biology Center, supported by the NIH Common Fund Transformative High Resolution Cryo-Electron Microscopy program (U24 GM129539), and by grants from the Simons Foundation (SF349247) and NY State Assembly.

## Conflict of interest

The authors declare that the research was conducted in the absence of any commercial or financial relationships that could be construed as a potential conflict of interest.

## Publisher’s note

All claims expressed in this article are solely those of the authors and do not necessarily represent those of their affiliated organizations, or those of the publisher, the editors and the reviewers. Any product that may be evaluated in this article, or claim that may be made by its manufacturer, is not guaranteed or endorsed by the publisher.
