# Supplementary material for: Analysis of two cooperating antibodies unveils immune pressure imposed on HIV Env to elicit a V3-glycan supersite broadly neutralizing antibody lineage
Source: Front Immunol. 2022 Sep 26;13:962939. doi: 10.3389/fimmu.2022.962939 (PMC9548623; doi:10.3389/fimmu.2022.962939)
Supplement: Supplementary file 1 [file DataSheet_1.docx]

Supplementary Material


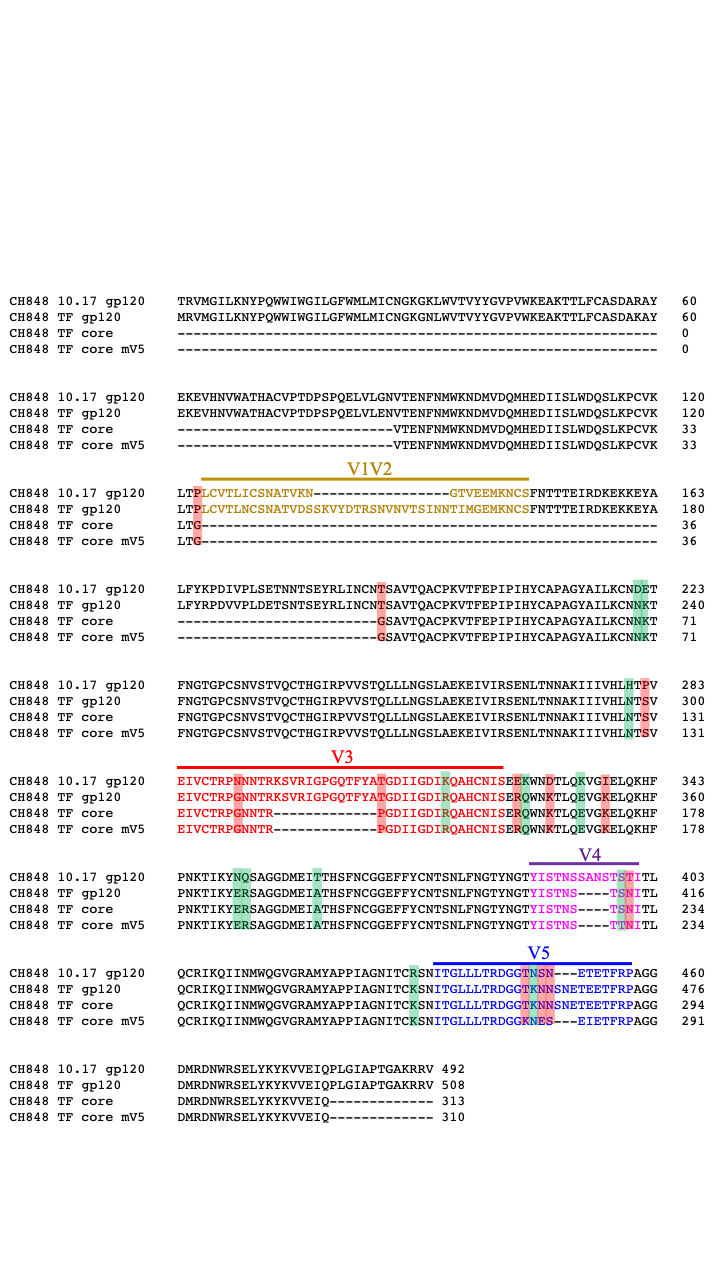


**Supplementary Figure 1.** Primary HIV Env constructs used in this study. Sequence alignment of the CH848.d0949.10.17 gp120 monomer, and the CH848 TF gp120, gp120 core and gp120 core mV5 constructs. Locations of variable loops are indicated (V1V2: gold; V3: red; V4: magenta; V5: blue). Differences between the sequences are highlighted, with conserved residues indicated in green and non-conserved residues indicated in red.


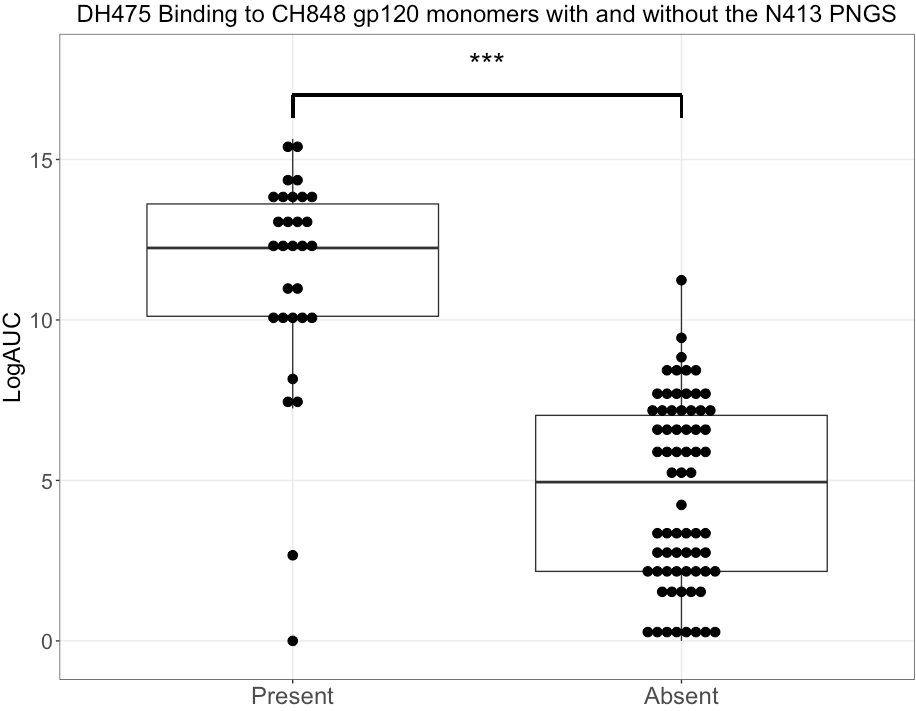


**Supplementary Figure 2.** Boxplot showing reduced DH475 binding affinity for CH848 virions without the N413 potential N-glycosylation site (PNGS) and various other mutations. Binding affinity is presented as logAUC from ELISA assays performed previously. Significant differences were found between groups (glycan present, n=30; glycan absent, n=69) using a Mann-Whitney U test, with *** indicating P<0.001.


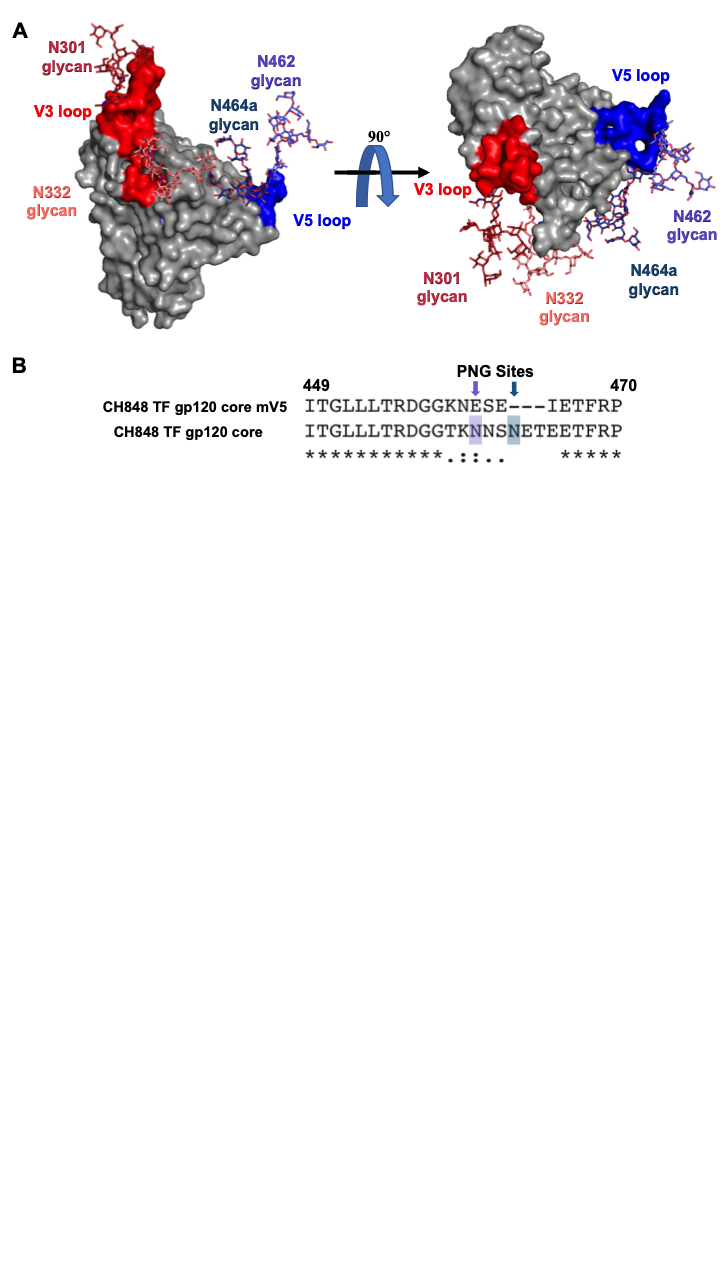


**Supplementary Figure 3.** Structural examinations of a V5 glycan epitope on the CH848 TF gp120 core. **(A)** Two views of a glycosylated CH848 TF gp120 core homology model (gray) with highlighted V3 (red) and V5 (blue) loops. V3 glycans N301 (light red), and N332 (pink), and V5 glycans N462 (lavender) and N464a (gray-blue) are shown as sticks and colored by atom (N: blue; O: red). **(B)** Comparison of V5 loop sequences of the CH848 TF gp120 core mV5 and gp120 core constructs, with potential N-glycosylation (PNG) sites at positions N462 and N464a highlighted.

**
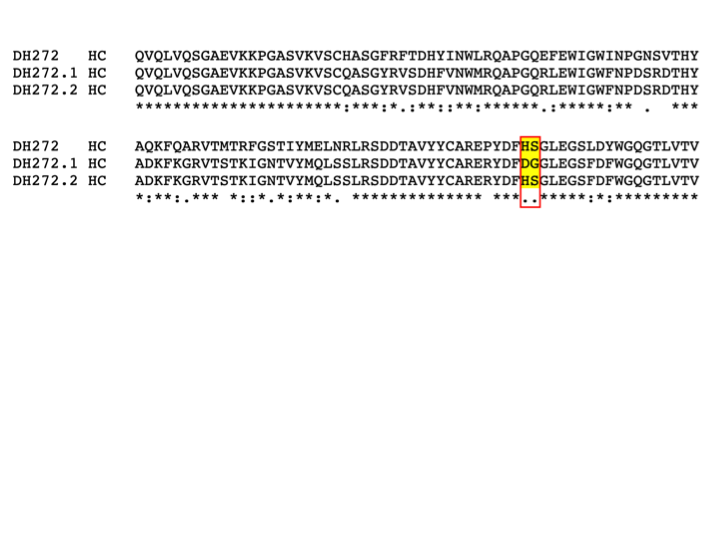
**

**Supplementary Figure 4. Protein sequence alignment of DH272 heavy chain (HC), DH272.1 HC, and DH272.2 HC variable domains**. Residues mutated to develop DH272.2 are highlighted. Asterisks (*) below the alignment indicate identical residues, colons (:) indicate conservative mutations, periods (.) indicated semi-conservative mutations, and spaces ( ) indicate non-conservative mutations.


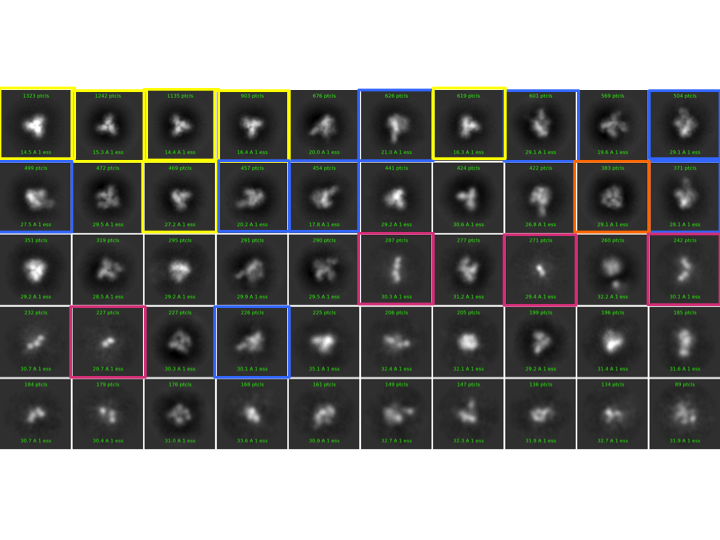


**Supplementary Figure 5.** **Negative stain EM 2D Class Averages of DH272.2 - CH848 TF DS SOSIP Env complex**. Representative ligands and complexes are boxed in different colors as follows: unliganded Env trimer (yellow), Env with one Fab bound (blue), Env with 3 Fabs bound (orange), and unbound Fab (pink).


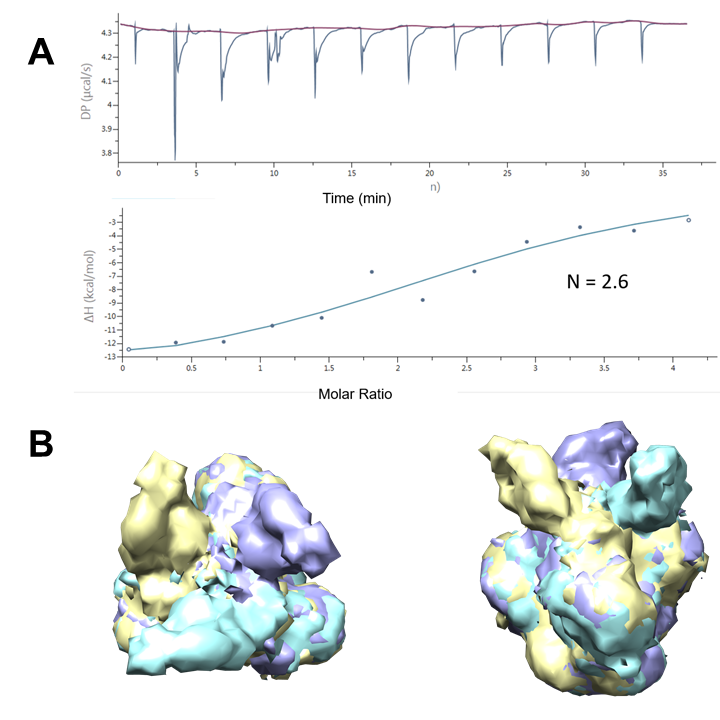


**Supplementary Figure 6.** Binding stoichiometry of DH272.2 with CH848 TF DS SOSIP Env. **(A)** ITC raw data (top) and analysis (bottom) for binding of DH272.2 with CH848 TF DS SOSIP Env. **(B)** Top view (left) and side view (right) of an alignment of three nsEM density maps (in cyan, purple, and yellow) such that the orientation of multiple Fabs bound to a trimer could be visualized relative to each other.

**Supplementary Table 1.** Data collection and refinement statistics

|  | DH475-Man_9_ |
| --- | --- |
| **Data Collection**  Space Group  **Cell dimensions**  a, b, c (Å)  α, β, γ (°)  Resolution (Å)  Total reflections  Unique reflections  Completeness (%)  Rmerge  I/σ(I)  Multiplicity  CC(1/2)  **Refinement**  Resolution  R-work / R-free (%) | P6_1_22  126.7 126.7 215.8  90 90 120  76.94 - 2.90 (3.004 - 2.90)*  22928  22831 (2202)  97.46 (96.75)  13.1  8.02 (0.87)  3.1  0.995 (0.545)  76.94 - 2.90  0.2291 / 0.2720 |
|  |  |
| **No. atoms** |  |
| Protein | 3295 |
| Ligand/ion | 127 |
| Water  Ramachandran favored (%)  Ramachandran outliers (%) | 65  96  0.23 |
| **R.M.S. deviations** |  |
| Bond lengths (Å) | 0.005 |
| Bond angles (°) | 1.09 |
| **B-factors (Å^2^)** |  |
| Protein | 82.60 |
| Man_9_ | 81.30 |
| Water | 73.20 |

**Values in parentheses are for highest resolution shell*
